# Earliest Cranio-Encephalic Trauma from the Levantine Middle Palaeolithic: 3D Reappraisal of the Qafzeh 11 Skull, Consequences of Pediatric Brain Damage on Individual Life Condition and Social Care

**DOI:** 10.1371/journal.pone.0102822

**Published:** 2014-07-23

**Authors:** Hélène Coqueugniot, Olivier Dutour, Baruch Arensburg, Henri Duday, Bernard Vandermeersch, Anne-marie Tillier

**Affiliations:** 1 Unité Mixte de Recherche 5199 – De la Préhistoire à l'Actuel: Culture, Environnement et Anthropologie (PACEA), Centre National de la Recherche Scientifique (CNRS) – Université de Bordeaux, Pessac, France; 2 Department of Human Evolution, Max Planck Institute for Evolutionary Anthropology, Leipzig, Germany; 3 Laboratoire d'Anthropologie biologique Paul Broca, Ecole Pratique des Hautes Etudes (EPHE), Paris, France; 4 Department of Anthropology, University of Western Ontario, London, Ontario, Canada; 5 Department of Anatomy and Anthropology, Sackler School of Medicine, Tel Aviv University, Ramat Aviv, Israel; 6 Museum of Archaeology and Anthropology, University of Pennsylvania, Philadelphia, Pennsylvania, United States of America; University of Kansas, United States of America

## Abstract

The Qafzeh site (Lower Galilee, Israel) has yielded the largest Levantine hominin collection from Middle Palaeolithic layers which were dated to *circa* 90–100 kyrs BP or to marine isotope stage 5b–c. Within the hominin sample, Qafzeh 11, *circa* 12–13 yrs old at death, presents a skull lesion previously attributed to a healed trauma. Three dimensional imaging methods allowed us to better explore this lesion which appeared as being a frontal bone depressed fracture, associated with brain damage. Furthermore the endocranial volume, smaller than expected for dental age, supports the hypothesis of a growth delay due to traumatic brain injury. This trauma did not affect the typical human brain morphology pattern of the right frontal and left occipital petalia. It is highly probable that this young individual suffered from personality and neurological troubles directly related to focal cerebral damage. Interestingly this young individual benefited of a unique funerary practice among the south-western Asian burials dated to Middle Palaeolithic.

## Introduction

Relevant information about Middle Palaeolithic societies can be obtained from paleopathological investigations. Identification of skeletal abnormalities and degenerative joint disease, as well as evidence for bone lesions caused by trauma, can provide insights into the adaptation patterns and social behavior of these early nomadic hunter-gatherers. With regard to south-western Asia, the first pathological data, to our knowledge, were those brought in 1939 by McCown and Keith's original description of the Mount Carmel people. During the last three decades, new attempts emerged in the studies of near eastern fossil record, related to enrichment in the fossil hominin sample. In this perspective, fossil specimens have benefited from new paleopathological investigations.

Among Levantine Middle Palaeolithic hominins, evidence of cranial traumatic lesions was provided by McCown and Keith [1] in their description of the partial skeletons from the Skhul Cave. According to these authors, the Skhul 1 child exhibits a depressed area in the mid-line of the frontal bone nearby the glabellar region which was interpreted [1] (pp 309–310) as consequence of a blow. These authors also mentioned the presence of a perforation and fracture of the right temporal in the roof of the ear which could result from an impact. However, the paleopathological condition of these two cranial lesions remains unclear as the authors themselves concluded [1] that both injuries “.. were inflicted at death or not unlikely at some time soon after death”. In an unpublished study, three of us (AmT, HD and BA) were not able to conclude if frontal and temporal changes observed on this fossil were pathological or taphonomical. McCown and Keith [1] (p 281) also drew attention to the presence of an injury “caused by a glancing blow at, or soon after death” in the left parieto-occipital area of the Skhul IX adult skull.

Later, in his original study of the Shanidar hominins from Iraqi Kurdistan, Trinkaus [2] provided a description of several pathological conditions displayed by one of the individuals, Shanidar 1. This adult individual sustained, among several skeletal lesions, a crushing skull fracture which involved the frontal process of the left zygomatic bone and the lateral margin of the left orbit. This ante-mortem traumatic injury most probably caused blindness of the left eye [2].

Within the Qafzeh hominin sample from lower Galilee, the skull of the adult Qafzeh 6 shows a concave indentation of the outer table of the frontal bone, without a fracture, in the area of the left supra-orbital region [3]. Such a condition can result from either trauma due to an accidental self-hurt or a blow to the head due to inter-personal violence. One of the immature individuals from the site, Qafzeh 11, presents a skull lesion previously attributed to healed trauma [4]–[6]. The goal of this study is to reappraise the Qafzeh 11 impact wound using 3D imaging methods, to better understand the pathological condition that affected this young individual. Indeed, 3D reconstructions applied to paleopathology allow us to better explore inner bone lesions, to evaluate their impact on soft tissues and to estimate volumetric data contributing to fossil reconstruction and preservation [7]–[9].

## Material

The Qafzeh site has yielded the largest hominin collection (N = 27, including partial eight skeletons, isolated bones and teeth) from Middle Palaeolithic layers in south-western Asia (*e.g.* [6], [10]–[11]). The Middle Palaeolithic sequence (units XVII to XXIII) was dated by a combination of electron spin resonance and thermoluminescence methods to *circa* 90–100 kyrs BP or to marine isotope stage 5b–c [12]–[13]. Human remains were discovered at the front of the cave's entrance in layers that contain a low density of lithic artifacts, a huge assemblage of micromammals and a few hearths. Within the Mousterian lithic assemblage [14]–[16], centripetal and/or bi-directional preparations prevail and the typical products are side scrapers, large oval flakes and quadrangular Levallois flakes. The makers of the Mousterian lithic industries at Qafzeh are identified as early anatomically modern humans [6], [10], [17].

A majority of the Qafzeh individuals fails to attain reproductive adulthood and among them, Qafzeh 11 is of special interest. It represents a single specimen recovered from layer XXIII, at the bottom of the Mousterian sequence, while most of the fossil human sample originates from layer XVII. A large stone damaged the trunk, pelvic area and lower limbs. Age at death of Qafzeh 11 was estimated *circa* 12–13 yrs while the sex remains unknown [5]. The partial skeleton of Qafzeh 11 is characterized by a combination of morphological traits in which modern features prevail, in comparisons with other Palaeolithic children [5]–[6]. Cranial morphology shows changes affecting the vault symmetry and base angulation; however their interpretation in terms of peri- or post-mortem changes remains unclear [6].

**Figure 1:**
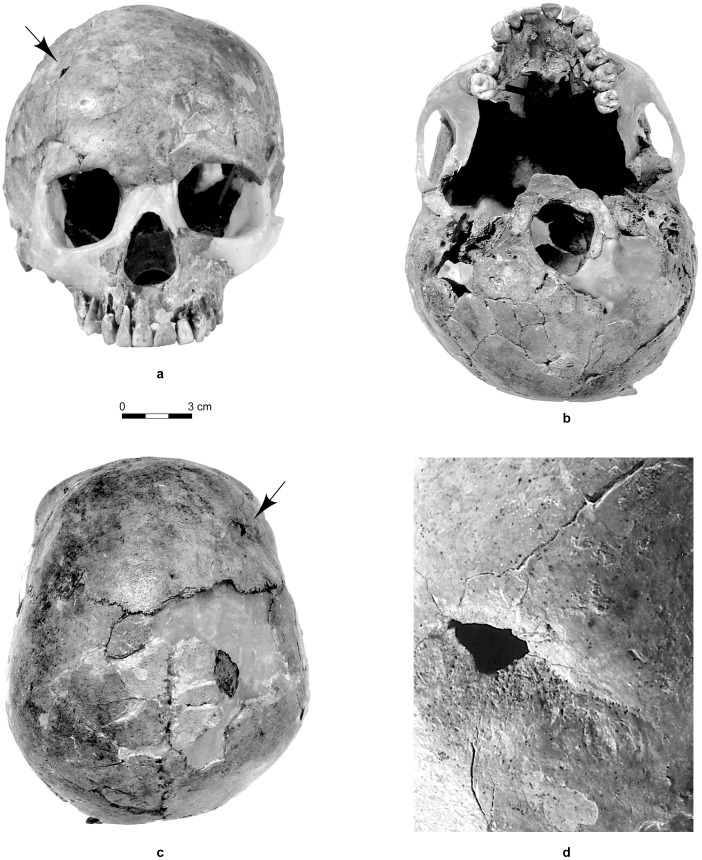
The Qafzeh 11 skull. a: norma facialis. b: norma inferior. c: norma superior. d: close-up view of the frontal lesion (healed fracture line is visible on the right side of the hole while fracturing lines above and below the hole are corresponding to post-mortem alteration). Black arrows on a and c indicate location of the lesion.

Besides these changes, Qafzeh 11 presents a cranial lesion previously attributed to a healed trauma [4]–[5]. This lesion is characterized by an anterior depression on the right side of the frontal squama. It is limited forwards by a healed fracture line, which ends up to an oval shaped hole. The latter has been attributed to a taphonomical change [4]–[5]. Healing process led to small thin bone remodelling, the frailty of it explaining its post-mortem loss (figure 1). Regarding the overall shape of the bone lesion and x-ray examination, the diagnosis of traumatic skeletal injury indeed prevails over that of an epidermoid bone cyst [3]. Surprisingly, comparative analysis between Qafzeh 11 and another child from same site, Qafzeh 10 younger in individual age (*circa* 6 years old), reveals that Qafzeh 11 had the smallest endocranial volume, respectively 1273±48 cc and 1251±48 cc [6].

## Methods

### Specimen number

Q11

### Repository information

Department of Anatomy and Anthropology, Faculty of Medicine, Sackler School of Medicine building Tel Aviv University, Ramat Aviv, Israel.

### Authority giving permission of study

Professor Israel Hershkovitz, curator of the collection, head of the Department. Last author (Am.T.) obtained his authorization for studying all the immature individuals from the Qafzeh site. The curator of this collection does agree the publication. There is no permit number.

Endocranial volume (EV) was estimated to set Qafzeh 11 within a normal modern variability of brain size growth, using two methods. EV was firstly calculated using equations recently proposed [18]; then an attempt of direct EV measurement on virtual endocast (see below) was performed although the skull base is damaged. For comparison, we used a modern data set issued from a digital bone library of immature skulls [18]. This sample comes from the identified osteological collection of Strasbourg University, France [19]. The EV values of Qafzeh 11 were compared to those available for other specimens (adult and immature) from the same site based upon cranial dimensions of Qafzeh 6, 9 and 10 [6], [10].

Following advances provided by digital 3D reconstructions, CT-scans of the Qafzeh 11 skull were carried out to reassess the traumatic condition which affected the adolescent during his/her life. These 3D reconstructions of Qafzeh 11 allow: (i) to precisely visualize 3D aspects of the internal and external surfaces of the cranial vault and of inner structures in the area of the pathological condition, (ii) to evaluate the potential impact of skull damage on the brain and (iii) to localize this impact on the brain surface.

Qafzeh 11 skull was CT scanned at the Carmel Medical Center, Haifa, Israel on a Brillance iCT 256, Philips Medical system (Cleveland, Ohio) with an isometric voxel size of 0.67 mm. Other acquisition parameters are 120 kV for voltage and 298 mA for current.

3D reconstructions of skull and endocast were performed using TIVMI software program [20] that is based on HMH (Half Maximum Height) algorithm [21] and applied to bone 3D reconstructions [22]. It has proved to be more precise and reliable for 3D measurements than other software programs currently implementing different algorithms for 3D reconstructions [23]. Besides providing additional estimation of the endocranial volume, virtual reconstruction allowed us to localize the impact of the cranial lesion on the brain surface, taking as a reference a 3D reconstruction of extant human brain [24] and checking the accurate correspondences of their anatomical landmarks [25].

Measurements of the endocast were taken using these landmarks and metric tools implemented in TIVMI. The cranium was horizontally oriented according to the “mean transverse plane” adapted from the original Frankfurt plane to study morphometry on digitalized skulls [26]; mean sagittal and coronal planes were drawn according to this method.

## Results

The endocranial volume of Qafzeh 11 ranges from 1283.44 to 1333.18 cc using updated formulas [18]. Previously, values ranging from 1251±48 cc to 1303±46 cc [6] were obtained using other equations [27]. EV value obtained from virtual endocast is slightly lower (1200 cc), but as anterior part of the skull base is missing, the endocranial virtual reconstruction is not complete.

**Figure 2:**
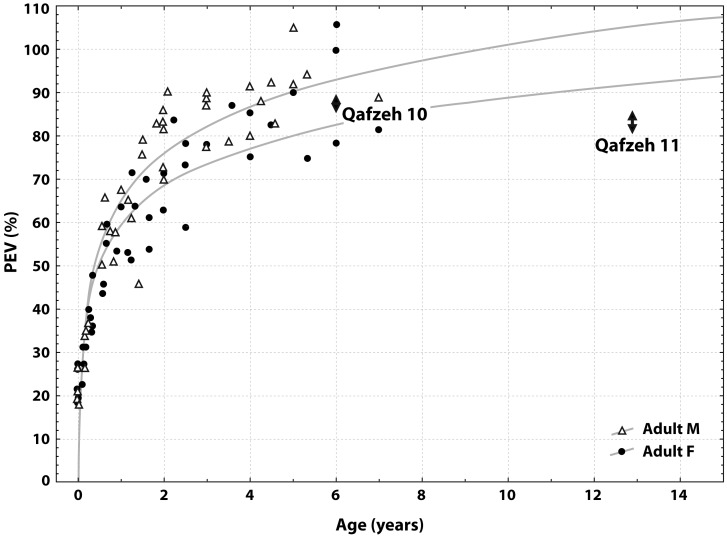
Proportional endocranial volume (PEV) of specimens Qafzeh 11 and 10 plotted on modern PEV from identified immature osteological collection (Coqueugniot and Hublin, 2012). Arrows represent PEV variation range.

The proportional endocranial volume (PEV) of Qafzeh 11, based upon EV values calculated from recent formulas, corresponds to 81–86% of the EV values of mature individuals from the site (Qafzeh 6 and 9). When considering dental maturation of this individual (giving an age estimation of about 12–13 years), this PEV value is smaller than expected in comparison with modern endocranial growth pattern defined by Coqueugniot and Hublin [18]. PEV value of Qafzeh 11 actually corresponds to values from younger children (4–6 years). For the same site, the PEV value of Qafzeh 10 child (estimated to be 6 years old from dental maturation) falls within the present modern range (86–88%) for the corresponding age (figure 2). Therefore, it appears that the small endocranial volume of Qafzeh 11 cannot be considered as normal relative to its dental age. Growth retardation in cranio-encephalic development can be proposed from this result.

**Figure 3:**
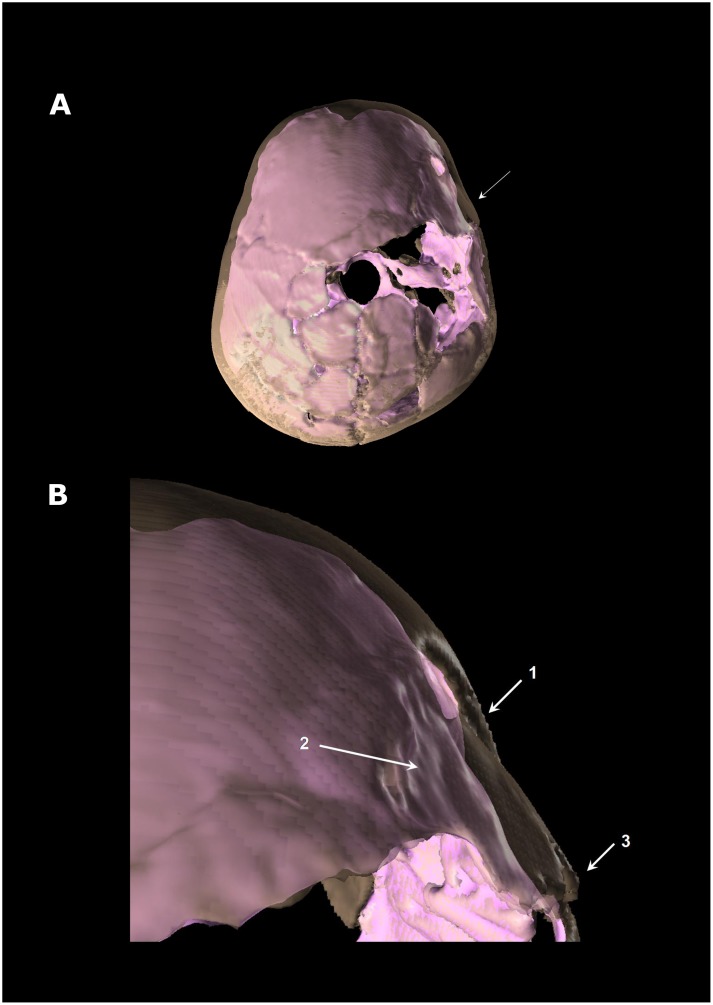
Superior view of Qafzeh 11 3D reconstructed skull showing the depressed fracture on the frontal's right side. The skull vault appears in transparency and the virtual endocranial cast in pink. A: general view. B: close up view of the trauma area. 1: anterior part of the frontal bone depressed fracture penetrating the endocranial volume. 2: irregular shape of virtual endocranial surface indicating brain damage. 3: diastasis of the right coronal suture.

The 3D reconstructed calvaria clearly evidenced a depressed skull fracture of the right part of the frontal bone in the process of healing (figure 3A). It is located on the right part of the frontal squama just above the pterionic area. The depressed fragment of the frontal bone has a quadrangular shape (size: 29.7×23.4×26.5×11.7 mm); the posterior face is delineated by the coronal suture, the anterior face is near the frontal boss as well as the upper face which lies at 31.8 mm from the frontal midline. The fractured fragment is depressed forwards. Its anterior face penetrates endocranially whereas the posterior face is shifted outwards, dislocating the coronal suture and causing a sutural separation (figure 3B). Other fracture lines, different from taphonomic fracturing, can be identified although they are less obviously visible due to the bone remodelling of healing process. This is shown by a star-like aspect of fracture lines radiating from the impact area. This type of fracture, that can be related to a blunt force trauma, clinically corresponds to cranio-encephalic wound and raises the question of its impact on the brain.

**Figure 4:**
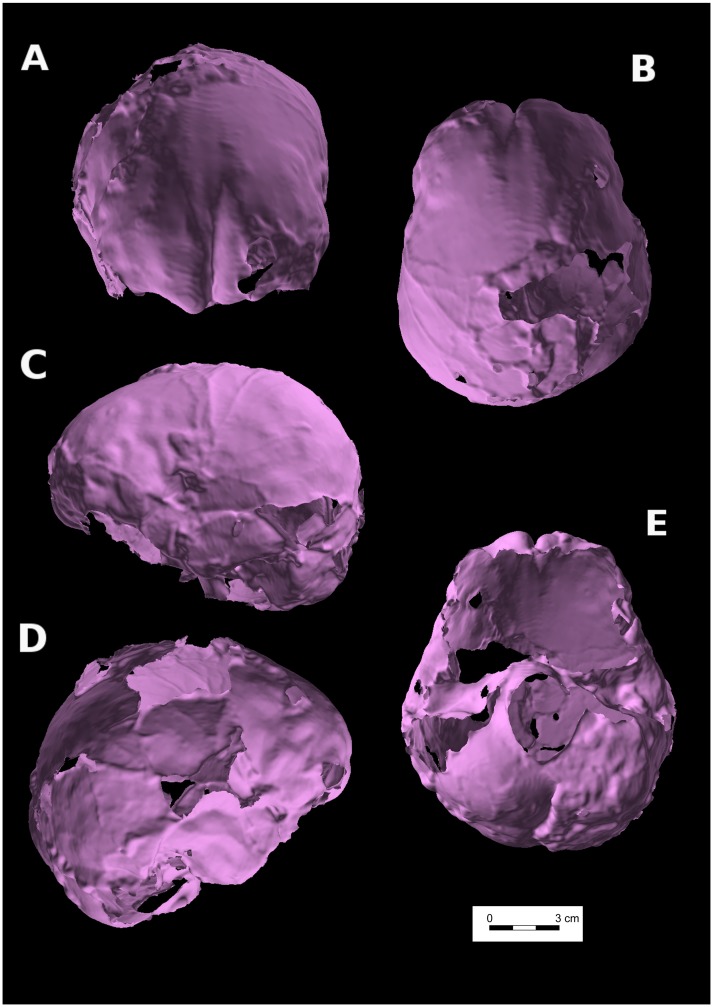
Virtual endocast of Qafzeh 11. A: norma frontalis. B: norma superior. C: left norma lateralis. D: right norma lateralis. E: norma basilaris.

Anatomical structures can be identified on the virtual endocast of Qafzeh 11 reconstructed by one of us (HC), despite missing parts, taphonomic fragmentation and post-mortem skull deformation (figures 3, 4). Normal cerebral hemispheres display an asymmetric development (figures 4A,B,E) characterized by a differential protrusion of one hemisphere relative to the other, known as petalia [25], [28]. The right frontal lobe protrudes in front of the left by 1.71 mm. In addition the right frontal bec is more extended downwards than the left one. By contrast, the left occipital lobe projects 1.79 mm behind the right one. Yet, the occipital asymmetry known as Yakovlevian torque [28]–[29] cannot be assessed here. Besides the right frontal petalia and left occipital one, the right frontal lobe is wider than the left and the left occipital lobe wider than the right. Interestingly, the two hemispheres are similar in length (153.7 mm at left and 153.6 mm at right).

Middle meningeal artery imprints are clearly visible on the left side of Qafzeh 11 endocast, showing the prevalence of the anterior branches (bregmatic and obelic) and lack of anastomosis (figure 4C), as previously described [6]. The imprint of the inferior frontal gyrus is visible on both sides. On the left it is possible to distinguish its reliefs (pars triangularis, pars opercularis and pars orbitalis) as well as those of the middle frontal gyrus and anterior central gyrus. On the right side, the imprint of the depressed fracture is localized upwards to the imprint of the inferior frontal gyrus. It may involve the posterior part of the middle frontal gyrus and the anterior part of the anterior central gyrus.

Comparison of the fossil virtual endocast with 3D reconstruction of digitized brain [24], confirms that the cranial depressed fracture observed on Qafzeh 11 (figures 3, 4D) only corresponds to the frontal area of the brain, forward to the central sulcus (Rolandic fissure) and upwards from the Sylvian fissure. The depressed skull fracture is localized forward to the precentral gyrus (primary motor cortex) and slightly behind the prefrontal cortex. The depressed fragment stretches over the middle part of the three frontal gyri. The corresponding brain areas are responsible for psychomotricity i.e. Brodmann areas 6 and 8 [30]. These areas control movement, rules for performing specific tasks, management of uncertainty, visual attention and eye movements [31]. The lesion may have affected the orbital part of inferior frontal gyrus (area 44) that is involved in speech language production on the left side (Broca's area), but seems to be involved in social communications on both sides.

## Discussion

When a pathological condition is recognized in skeletal remains, the nature of the bone damage or injury is sometimes not easy to determine precisely. Peri-mortem trauma can be difficult to differentiate from skeletal post-mortem changes due to taphonomic processes (e.g. [32]). Cases of serious cranial trauma are seldom documented in the human Upper Pleistocene fossil record from south-western Asia (e.g. [2]–[5]) and Western Europe [33]–[34]. Zollikoffer et al. [33] asserted that the cranial injury displayed by the Neanderthal St-Césaire 1 resulted from an act “of intragroup, interpersonal violence” but did not cause the immediate death. Examining the pathological condition of Krapina 34.7 parietal fragment, Mann and Monge shared the same statement, i.e. the serious trauma “was not a mortal wound”; however they concluded that its cause “appears to one of an accident associated with life style of living and sleeping in caves” [34].

In his original description of the healed trauma which affected Qafzeh 11, Dastugue [4] mentioned that the skull fracture was not lethal, related to a minor trauma and only localized on the skull vault. According to him, this so-called “benign fracture” did not have significant repercussions and occurred when Qafzeh 11 was young. Furthermore, the healing response had probably not undergone its complete trajectory before the death of the adolescent [4]. Dastugue concluded that the cause of death was unknown.

3D reconstructions clearly show that the Qafzeh 11 skull fracture was not a simple one. Indeed, this frontal bone fracture appears to be compound, with a broken piece of frontal squama that is depressed, isolated forwards by a linear fracture and backwards by sutural diastasis. As previously mentioned [4], this fracture type generally results from a blunt force trauma (getting struck or kicked in the head by heavy and blunt material, accidentally or intentionally with weapon). This type of trauma can be interpreted as resulting from interpersonal violence, but as has been demonstrated by paediatricians, complex cranial fractures like this one can also occur accidentally [35]. Contrary to the assumption of a non-serious wound made previously [4], the depressed fracture of Qafzeh 11 skull that can be considered as at least a moderate traumatic brain injury (TBI) [36], actually presents a high level of risk for brain damages (intra-cranial haemorrhages, diverse types of central nervous system lesions such as concussion, contusion, laceration, which can lead to destruction of brain tissue or cerebral scar). Besides the neurological damages due to focal brain lesion in the right frontal area, more precisely the areas 6 and 8. These areas are responsible for psychomotricity which may have led to troubles for controlling movement, difficulties for performing specific tasks, managing uncertainty, visual attention and eye movements and possibly the right area 44 (that seems also to be involved in oral communication as the left Broca's area, that is specialized in speech production). It is highly probable this young individual suffered also from personality changes due to traumatic brain injury. This personality disturbance is thought to be directly related to brain trauma and appears to be very frequent: 65% in severe to mild/moderate TBI, according to Max et al. [36]) but according to McAllister [37] “virtually all individuals who survive moderate and severe TBI are left with significant long-term neurobehavioral sequelae”. These troubles are characterized by a “distress or impairment in social, occupational, or other important areas of functioning” and manifested in children as a “marked deviation from normal development” [36].

Two methods have confirmed the small endocranial volume of Qafzeh 11. The virtual reconstructed endocranial volume provides an underestimated value due to the lack of anterior part of skull base which technically limits endocast segmentation and therefore makes its complete virtual reconstruction speculative. Recently, Kondo et al. [38] proposed a semi-virtual reconstruction of the Qafzeh 9 endocast. They obtained a EV value of 1411–1477 cc that is smaller than the initial estimation of 1508–1554 cc [10] and the mean value of 1531 cc provided by Holloway et al. [25]. Considering that (i) EV virtual values appear to be smaller than calculated ones for the base- damaged Qafzeh 9 and 11 skulls, (ii) virtual EV are not available for other specimens of the site (Qafzeh 10 and 6), we prefer using estimated EV value calculated from formulae.

As for Qafzeh 11, EV values are nevertheless consistent each other and corroborate a small endocranial volume related to individual age whatever the method used. This can be interpreted as growth retardation due to the trauma. Indeed, generalized atrophic changes resulting in reduced overall brain volume has been documented in moderate-to-severe pediatric traumatic brain injury [39]–[40]. In addition to this focal effect on brain, a general growth retardation due to post-traumatic endocrine disturbance [41] could be raised here.

Hemispheric asymmetry is present on Qafzeh 11. This feature has already been described on fossil hominins (e.g. [25], [29], [42]–[43]) including Qafzeh 9 [38] and among extant populations (e.g. [28], [44]). Therefore, despite the depressed frontal fracture that had probably impacted the underlying brain tissue of Qafzeh 11 frontal lobe, the physiological hemispheric asymmetric pattern was not affected.

As Qafzeh 11 has a PEV corresponding to a 4–6 years old modern child, we hypothesize that the trauma occurred at or before this age. Among skeletal indicators of growth disturbance and stress during childhood, is the manifestation of growth arrest lines (Harris lines) in the metaphyseal region of the long bones. These non-specific stress indicators usually vanish during life. Unfortunately, the preservation state of long bones does not allow any kind of investigation in the case of Qafzeh 11. Pathological alterations of the dental enamel, such as transverse linear enamel hypoplasia (LEH), are also employed in the assessment of physiological stress events and growth disturbances during childhood (e.g. [45]–[48]). Presence of enamel hypoplasia on three lower teeth of Qafzeh 11 (right and left first molars, right second molar) was previously described by Skinner [49]. However, data collected on the specimen by one of us (AmT) point to the lack of LEH on the permanent upper and lower teeth and on the isolated germs of upper third molars as well [3]. Both lower right M1 and M2 indeed present a different enamel coloration above the cervix, located at the same height of the two crowns. This alteration is most probably of taphonomic origin and we suggest that the skull trauma didn't impact M1 and M2 complete crown formation, indicating that it probably occurred around 6 years of age.

In sum, the Qafzeh 11 child represents, to our knowledge, the oldest documented human case of severe cranial trauma available from south-western Asia, dated to 90–100 kyrs BP. The adult Shanidar 1 skull exhibits an indisputable evidence of trauma, that was sometimes interpreted as a consequence of interpersonal violence [2], [50] but the specimen is probably more recent [51]. For Qafzeh 11, the exact circumstances surrounding the injury remain unknown, although this kind of injury generally results from a blunt force trauma.

Whatever the origin and severity of a given pathological condition observed on human Middle Palaeolithic hominins, speculations were made with regard to its consequences on individual life conditions and social status, in terms of disability, impairment and social care. Consequently, these questions are widening the debate introducing notions of altruism and compassion in prehistoric human communities and their possible role in human life history (e.g. [2], [52]–[58]).

**Figure 5:**
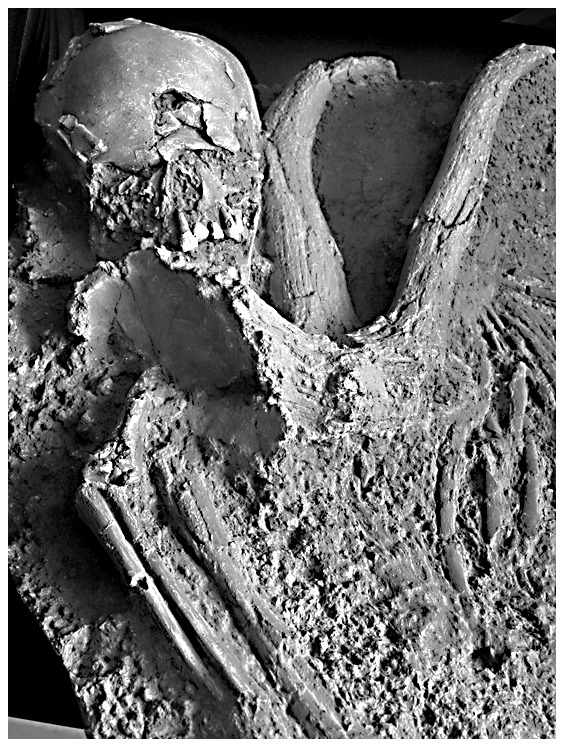
Partial view of the Qafzeh 11 burial showing the deposit of the red deer antlers in close contact with the child skeleton (cast).

In this respect, it is crucial to assemble biological and pathological data with cultural observations and their subsequent interpretations. For the Qafzeh 11 subadult, it is now clear that severe cranio-encephalic trauma experienced during childhood, deeply impacted his/her cognitive and social communication skills. Interestingly Qafzeh 11 benefited from special social attention at his/her death, as shown from archaeological details. The Qafzeh 11 skeleton, recovered at the bottom of the Mousterian sequence in front of the entrance of the cave, revealed that the corpse was originally lying in a pit on its back, the head turned to the right with upper limbs flexed [59]. The hands maintained their anatomical configuration and were lying together near the face western-oriented. The pelvic region and the lower limbs extended to the south from the skull, were post-depositionally damaged by a large stone. Besides this, there was a complete lack of mixing or bone displacement with an absence of animal scavenging traces. Furthermore, two deer antlers were lying on the upper part of the adolescent's chest, near his/her face and they were in close contact with the palmar side of the hand bones (figure 5). Such a hand location, within the original body spatial arrangement, attested a funerary offering and not an accidental incorporation. All these observations strongly support the interpretation of a deliberate, ceremonial burial for Qafzeh 11.

At Qafzeh several other burials occur [59]–[62], but Qafzeh 11 represents a unique case of differential treatment with convincing evidence for ritual behavior. We interpret the Qafzeh 11 burial as resulting from a ritual practice applied to a young individual who experienced a severe cranial trauma most probably followed by significant neurological and psychological disorders, including troubles in social communication. These biological and archaeological evidences reflect an elaborate social behavior among the Qafzeh Middle Palaeolithic people.
